# Comparison of electrode position marking procedures on the cranial surface

**DOI:** 10.1002/brb3.3187

**Published:** 2023-08-03

**Authors:** Albert Fabregat‐Sanjuan, Rosa Pàmies‐Vilà, Agnès Rigo‐Vidal, Vicenç Pascual‐Rubio

**Affiliations:** ^1^ FUNCMAT, Mechanical Engineering Department Universitat Rovira i Virgili Tarragona Spain; ^2^ BIOMEC, Mechanical Engineering Department Universitat Politècnica de Catalunya Barcelona Spain; ^3^ NeuroÈpia, Clinical Neurophysiology Department Institut d'Investigació Sanitària Pere Virgili, Hospital Universitari Sant Joan de Reus Tarragona Spain

**Keywords:** 10/20, cranial landmarks, evoked potentials, neurophysiology

## Abstract

**Introduction:**

The study aimed to compare the conventional method of electrode marking with a new system, EPlacement, to improve accuracy and reduce the time burden on health care professionals.

**Methods:**

Ten health care professionals marked mannequin heads and adult volunteers using both methods. Time, accuracy, and usability of each method were analyzed. Three neurophysiological diagnostic tests were performed on mannequin heads: reversal pattern visual evoked potential (three electrodes required); somatosensory evoked potentials from the upper and lower extremities (five electrodes required); and standard intraoperative neurophysiological monitoring for spine surgery (nine electrodes required). Precision scanning of the mannequins with structured light and a printed hull were used to determine the actual locations of the electrodes of the 10/20 system.

**Results:**

The new method based on the EPlacement device represents an improvement on conventional tape measure (TM) marking and may be considered within the group of advanced methods such as navigation systems since it leads to improvements of 34% (1.7 mm) for electrode positions in the Nasion‐Inion and Left tragus–Right tragus lines and 77% (12.5 mm) for electrode positions using the approximate method. It reduces the time spent per test by an average of 1 min compared to the TM method. Health care staff survey results show a positive feedback regarding usability of the new method.

**Conclusions:**

The study showed that the EPlacement device improves accuracy, reduces time, and is easy to use compared to the conventional method of electrode marking. The EPlacement method can facilitate the complex task of electrode marking and ultimately contribute to improved patient outcomes. It has the potential to be widely accepted and implemented in clinical practice.

## Graphical abstract

New method using EPlacement simplifies and improves the accuracy of the marking process, and the time burden from clinical staff. EPlacement does automatic calculations as well as detailed guidance on indicating the electrode positions required for each test. Results suggest that EPlacement could be a valuable tool for improving the accuracy and efficiency of electrophysiological tests.

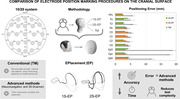

## 1 INTRODUCTION

Numerous neurophysiological tests (e.g., evoked potentials, electroencephalography [EEG], and noninvasive brain‐stimulation techniques) require recording or stimulation devices to be placed on the scalp close to the underlying cerebral cortex and subcortex.

Advanced methods, such as neuronavigation and real‐time 3D scanners (De Witte et al., 2018; Jog et al., 2021), can be used to correctly locate these cranial positions. However, these methods are not universally applied due to the cost, time, and complexity involved (Nieminen et al., 2022; Scrivener & Reader, 2022).

To position the devices, most electrophysiology laboratories use the 10/20 system developed by the International Federation in Clinical Neurophysiology (IFCN) (Seeck et al., 2017). This system works by dividing the cranial surface into 10% and 20% segments in order to locate the underlying cortical areas of interest. The conventional method for achieving this is to position a tape measure (TM) in line with the basic anatomical references of the head and to calculate distances to position the electrodes according to the methodology of the international 10/20 system (Jurcak et al., 2007).

The number of cranial points to be located depends on the type of test selected. Each point is highlighted with a marker pen (this is the *marking* procedure) so they can be visually detected by health care staff and the electrodes can be placed properly. This marking must be performed accurately so as to avoid erroneous or even counterproductive results and ensure the optimal application of the neurophysiological technique.

However, with this conventional method for locating cranial points in accordance with the 10/20 system, numerous sources of error can be introduced. Sometimes the percentage calculation is made under stressful conditions for health care staff (such as on a night shift in an intensive care unit with a high care load), which can lead to calculation errors. Other situations, such as intraoperative neurophysiological monitoring (IONM) (Nagle et al., 1996), require fast and accurate marking in order not to delay surgeries that may themselves be complex and time‐consuming. The electrodes must also be placed extremely accurately as they are usually spiral needle electrodes that remain in place for the duration of the surgery. Accurate placement avoids having to reposition the electrodes, which may entail interrupting the surgery or endangering the sterile conditions of the surgical field.

Moreover, certain target points are required for each test and, since both hands are busy holding the TM, individualized percentage calculations must be made mentally (Jurcak et al., 2007; Seeck et al., 2017). In addition, if the electrodes to be placed do not match the cranial perimeter or the Nasion‐Inion (Ns‐In) or Tragus–Tragus (LPA–RPA) planes (e.g., positions F3, F4, CP3, CP4, P3, and P4), the positioning error (PE) is larger because the locations of these electrodes are dependent on other positions established via previous measurement, which may cause the progressive accumulation of errors (Böcker et al., 1994; Mir‐Moghtadaei et al., 2015).

Several systems are available for placing or assisting in the placement of the electrodes. These include EEG caps. These possess already established electrode positions (Atcherson et al., 2007; Trapp et al., 2019) and are elastic so that they adapt to the patient's cephalic surface. However, they do not cover the whole range of head sizes (a range of caps exists to cover several but not all cranial perimeters) and suffer nonhomogeneous deformation (which is maximal in the temporal regions of the cap). Therefore, the actual positions often do not coincide with those of the 10/20 system. EEG caps are not normally used for electrophysiological tests other than EEG, either because the required number of electrodes is smaller (in the case of evoked potentials [Kovalova et al., 2016], for example) or because it is best not to cover the head during the test (for example, during the application of repetitive transcranial magnetic stimulation or during a EEG, where the fontanelles should be visible and access to the epicranial veins may be needed). In these cases, the electrodes are placed individually on the scalp.

To avoid these drawbacks, a marking tool is required that allows the clinical staff to be guided quickly and precisely during the placement of the electrodes on the cranial surface and that adapts to the requirements of each neurophysiological test. This is the aim of EPlacement, a new device developed by the research group responsible for this article. This new device solves the problems of conventional marking since it provides a fast, simple and accurate way to determine the location of cranial points in the context of tests and neurophysiological studies.

In this paper, we analyze the differences between the conventional marking method and EPlacement in terms of time, accuracy, and the opinions of health care staff.

## 2 MATERIALS AND METHODS

### 2.1 EPlacement

The EPlacement device consists of an electronic unit (a display, a microcontroller, and a battery), a pressure sensor, and an illumination system with a high density of LEDs. The electronic unit has a navigation menu from where the clinician chooses the test to be performed. For this study, the following tests were programmed: pattern‐reversal visual evoked potential, (PVEP), somatosensory evoked potentials (SEP) (Liu et al., 2017), and standard spinal IONM (Goryawala et al., 2012). The device provides a step‐by‐step guide to the different points required for each test. The pressure sensor and illumination system are embedded in a strip that easily adapts to the shape of the head.

The health care staff initially adjust the strip on the head and locate the cranial reference points in accordance with the 10/20 system (Nasion, Inion, or Tragus). The identification of reference points is performed manually by health care staff, but once these reference points are identified, the calculation is carried out automatically. The health care staff simply needs to press the strip on the reference point (the sensor automatically detects the pressure), and if the pressure is applied for more than 3 s, the measurements are automatically obtained. The touch sensor used in the system is a linear soft membrane potentiometer. When pressure is applied to the touch sensor, the resistance undergoes linear changes, enabling precise calculation of the relative position on the strip. The system directly measures the length of the head, whereas the microcontroller performs the percentage calculations and illuminates the LED that indicates the point to be marked on the scalp. The length of the touch sensor is designed to cover the majority of adult cranial dimensions, accommodating variations in head size.

**FIGURE 1:**
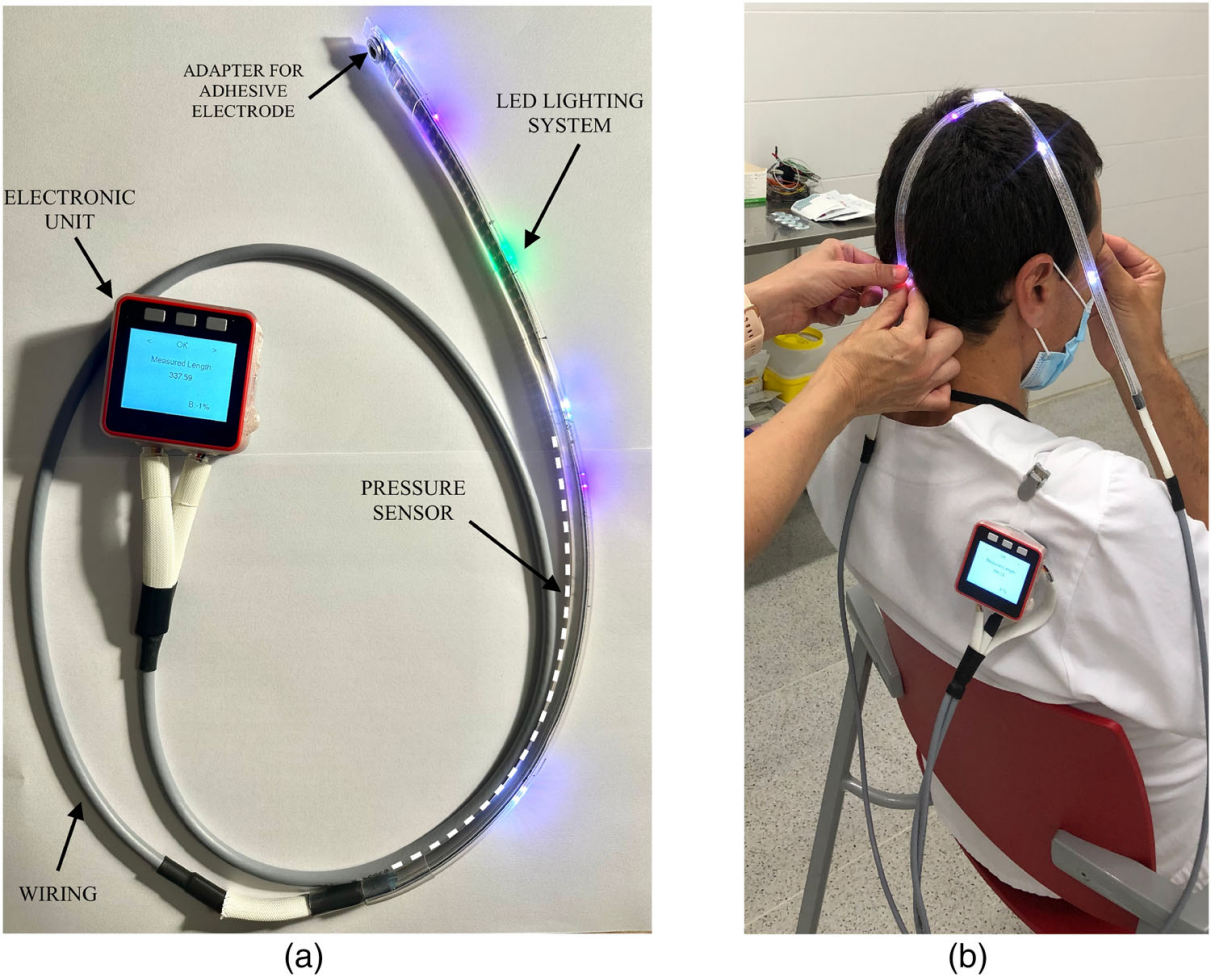
(a) EPlacement device and its components. (b) EPlacement device (two‐strip version) mounted on a volunteer.

We designed two different versions of the EPlacement device. The first version comprises a single strip (1S‐EP), whereas the second one comprises two strips that allow relative movement between them (2S‐EP). The navigation menu adapts the procedure to the version of the device being used. Figure 1a shows the device with one strip, and Figure 1b shows the device with two trips mounted on a volunteer. To explore the functionality of the device, the patent is available at (Fabregat‐Sanjuan & Pascual‐Rubio, 2022).

Typically, the marking process begins by obtaining the electrode positions relative to the midline of the head. For this purpose, in the case of the two‐strip configuration (2S‐EP), one strip is positioned to cover the tragus reference points, whereas the second strip is placed over the Inion and Nasion reference points. The microprocessor calculates the distances and illuminates midpoints where the strips have to intersect. Once the strips are appropriately positioned, the desired test can be conducted.

In the single strip arrangement (1S‐EP), an additional step is required to establish the midline compared to the two‐strip version. The user initiates the process by measuring the LPA–RPA distance. Once this measurement is obtained, the device illuminates a point along the midline that needs to be marked. Subsequently, the user measures the Ns‐In distance, passing through the Ns and the previously marked point, and the device illuminates the accurate Cz position. The midline is determined based on the Ns‐In and Cz points.

### 2.2 Study description

To conduct this study, authorisation was received from the Ethics Committee of the Pere Virgili Institute for Health Research (IISPV).

Participants were 10 health care professionals from the Hospital Universitari Sant Joan de Reus (HUSJR). The study comprised two parts. In the first part, the health care professionals carried out markings on mannequin heads, and the time and accuracy of each marking method were analyzed. In the second part, these clinicians marked the heads of adult volunteers so as to evaluate the usability of EPlacement on actual human heads. In this case, however, on account of the large variability in hair density, hair length, and head shape, neither time nor accuracy was analyzed. The opinions of the clinicians were collected by means of a survey.

With regard to the markings on the mannequin heads, three neurophysiological diagnostic tests were analyzed: reversal PVEP (three electrodes required) (Schoenen et al., 1995); SEP from the upper and lower extremities (five electrodes required) (Cruccu et al., 2008); and standard IONM for spine surgery (nine electrodes required), which comprised the same electrodes as SEP but also included motor evoked potentials for stimulation purposes.

Table 1 shows the setup for the neurophysiological diagnostic tests. ON stands for optic nerve, UL stands for upper limb; and LL stands for lower limb. Table 1 also shows the active electrode (Act.), reference electrode (Ref.), and ground electrode (Gnd.) for each test except for C3–C4 and C1–C2 that are cathode and anode.

**TABLE 1:** Setup of the three electrophysiological tests.

		IONM (9E)			
	VEP (3E)	SEP (5E)		MEP (4E)	
	ON	UL	LL	UL	LL
Act.	Oz	CP3/CP4	CPz	C3–C4	C1–C2
Ref.	Fpz	Fz			
Gnd.	Cz	Cz			

Abbreviations: IONM, intraoperative neurophysiological monitoring; SEP, somatosensory evoked potentials.

Each test was performed three times, each time with a different tool, that is, single‐strip EPlacement (1S‐EP), double‐strip EPlacement (2S‐EP), and TM.

The clinicians performed each electrophysiological test on a different mannequin head (a total of 30 mannequins were used). As each test was conducted three times, the first and second markings (using EPlacement) were performed with two markers visible only with ultraviolet light (blue and pink) so as not to influence the third marking (TM), which was performed with a black marker. Each marking process was timed.

Note that, in clinical practice, when the TM is used for the CPz, CP3, and CP4 positions, clinicians do not usually place the electrodes according to the international 10/20 system. Instead, they use an approximate method to place the above electrodes 2 cm behind the Cz, C3, and C4 electrodes, which are renamed respectively: Cz′, C3′, and C4′ (Dumitru et al., 2002; Nuwer, 1987). Our study has been conducted in accordance with this usual practice. For this reason, in this study, the electrode positions were divided into two groups: those on the Ns‐In and Tragus–Tragus lines (NITT) and those made according to the Approximate method used in Clinical Practice (ACP) (Ozkul & Uckardes, 2002) (in our case, CPz, CP3, and CP4).

In the second part of the study, the suitability and ease of use of each method were assessed by means of a survey.

For this purpose, the markings on the volunteers’ heads were the same as for the upper‐ and lower‐limb SEP (five electrodes).

**FIGURE 2:**
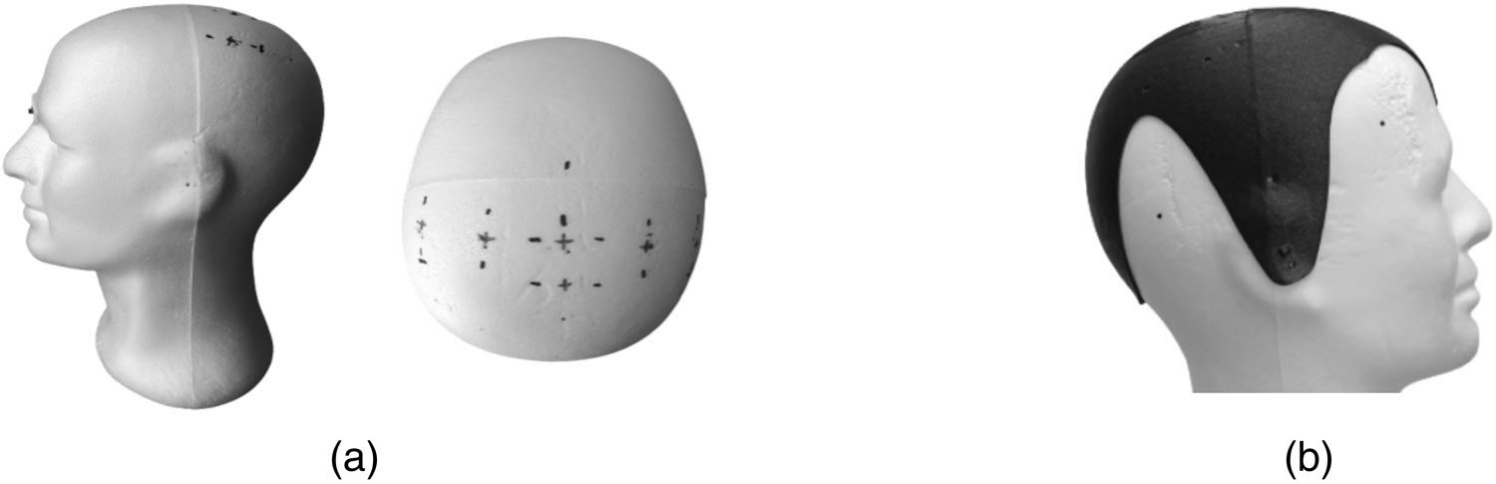
(a) Mannequins with markings. (b) Printed template mounted on the mannequin.

Precision scanning of the mannequins with structured light (HP 3D Pro S3) was performed. The resulting surface was imported into Autodesk Fusion 360 software, which was used to determine the actual locations of the electrodes of the 10/20 system. These locations were found using the method proposed by the system (Balolia & Massey, 2021) from the Ns‐In and LPA–RPA measurements, which were obtained by intersecting the plane with the corresponding section. With the same software, a 2 mm thick hull/template with holes perpendicular to the surface was created to locate the positions of the 10/20 system (Figure 2b). The hull was printed on an Original Prusa i3 MK3S+ printer.

As explained earlier, 30 identical mannequin heads were used for this study. On each mannequin, once the manual markings were made, the 3D printed helmet was attached and the reference positions of the 10/20 system were marked. Using ultraviolet light, the points of the first and second markings (obtained with the two versions of the EPlacement device, Figure 2a) were then marked with needles. Finally, for each position of the 10/20 system, the distances between the experimental points (marked using EPlacement devices and the TM) and the reference positions (obtained from the template helmet) were measured using a digital calliper (0.01 mm resolution).

### 2.3 Error statistics

PE was measured by obtaining the Euclidean distances between the positions of the template helmet and the positions of the marking procedure. For each electrode, *i* (*i* = Fpz, Fz, Cz, Oz, C3, C1, C2, C4, CP3, CPz, and CP4), a set of distances were obtained depending on the tool used (*j* = 1S‐EP, 2S‐EP, and TM): ${\mathrm{PE}}_{i}^{j}$. For each method and each electrode, the average (${\bar{\mathrm{PE}}}_{i}^{j}$.) and standard deviation of the PE were calculated (${\mathrm{sd}}_{i}^{j}$). Because some electrodes were used in more than one neurophysiological test, the number of PEs of each electrode (sample size) was not constant. The standard error ($se_{\bar{j}}$) was calculated by dividing the standard deviation of the sample by the square root of the sample size.

A Tukey HSD test was performed to determine whether the averages of the errors obtained with the three tools for the two electrode positioning groups were statistically different. This test performs a multiple comparison between all pairs to detect whether the PE obtained has a statistically significant mean difference with a *p*‐value <.05.

To compare the differences in the times used to position the electrodes, another Tukey HSD test was performed. In this case, the time differences among the three tools were analyzed. Again, a *p*‐value below .05 indicates that significant differences in mean times exist among the tools.

## 3 RESULTS

### 3.1 Marking accuracy

**FIGURE 3:**
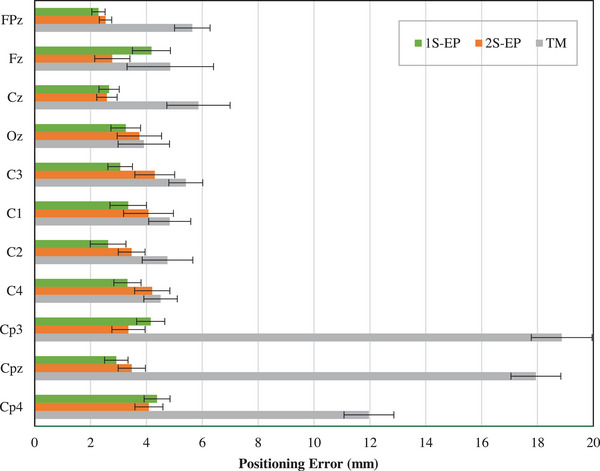
Positioning error per electrode depending on the tool used: 1S‐EP (green), 2S‐EP (orange), and tape measure (TM) (gray). Mean positioning error ± standard error.

Our results are shown in Table 2, which lists the sample size, mean error, standard deviation, and standard error for each electrode and tool used. Figure 3 shows a grouped bar graph of the mean distance error per electrode for each type of marking: 1S‐EP (green), 2S‐EP (orange), and TM (gray). Each bar represents the overall mean value of the error at each position ± the standard error.

**TABLE 2:** Positioning errors (PE) for each electrode position depending on the tool used (1S‐EP, 2S‐EP, and tape measure [TM]): Sample size (*n*), mean error (${\bar{\mathrm{PE}}}^{j}$), standard deviation (sd^*j*^), and standard error $(se_{\bar{j}}).$

		EP1			EP2			TM		
	*n*	${\bar{\mathrm{PE}}}^{\mathrm{EP}1}$	sd^EP1^	$se_{\bar{\mathrm{EP}1}}$	${\bar{\mathrm{PE}}}^{\mathrm{EP}2}$	sd^EP2^	$se_{\bar{\mathrm{EP}2}}$	${\bar{\mathrm{PE}}}^{\mathrm{TM}}$	sd^TM^	$se_{\bar{\mathrm{TM}}}$
FPz	30	2.282	1.301	.238	2.535	1.222	.223	5.642	3.485	.636
Fz	10	4.178	2.150	.680	2.778	1.996	.631	4.853	4.896	1.548
Cz	20	2.663	1.623	.363	2.585	1.633	.365	5.863	5.062	1.132
Oz	10	3.260	1.678	.531	3.747	2.512	.794	3.905	2.912	.921
C3	10	3.060	1.394	.441	4.298	2.257	.714	5.409	1.919	.607
C1	10	3.348	2.069	.654	4.074	2.822	.893	4.834	2.384	.754
C2	10	2.628	2.021	.639	3.470	1.517	.480	4.755	2.865	.906
C4	10	3.320	1.538	.486	4.208	1.994	.631	4.505	1.893	.599
Cp3	20	4.152	2.255	.504	3.358	2.660	.595	18.867	4.864	1.088
Cpz	20	2.920	1.878	.420	3.476	2.176	.487	17.944	3.992	.893
Cp4	20	4.381	2.073	.464	4.088	2.243	.502	11.967	3.991	.892

These results illustrate the importance of grouping the electrodes according to those that lie on the Ns‐In and LPA–RPA lines (NITT) and those that do not (ACP group). For the NITT electrodes, the average errors were 5.0, 3.1, and 3.5 mm with the TM, 1S‐EP, and 2S‐EP, respectively. For the ACP electrodes, a larger error was observed with the TM (average error = 16.3 mm) while the errors observed with the EPlacement devices were similar to those for the NITT electrodes (PE = 3.8 mm for 1S‐EP and PE = 3.6 mm for 2S‐EP).

**FIGURE 4:**
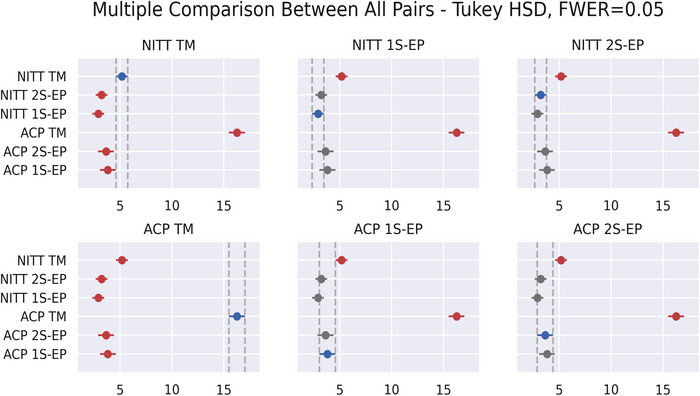
Tukey HSD test of the positioning errors for the tools (tape measure [TM], 1S‐EP, and 2S‐EP) and electrode groups (ACP and NITT).

To evaluate the significance of these differences, Figure 4 shows the multiple comparison between all pairs (Tukey HSD test). The results indicate that the mean PE with the TM is always significantly greater than with the EPlacement devices. Note that these differences are observed for both the ACP electrodes and the NITT electrodes. On the other hand, no differences are observed between the two versions of the EPlacement device.

### 3.2 Timing

The times taken to perform each neurophysiological test (PVEP, SEP, and IONM) were measured. Table 3 shows the average times, standard deviations, and standard errors for each tool. Differences are observed between the TM and EPlacement devices for all three tests. On the other hand, the performances of both EPlacement devices were similar.

**TABLE 3:** Average time (AT) needed to conduct the neurophysiological tests depending on the tool used (1S‐EP, 2S‐EP, and tape measure [TM]), sample size (*n*), average time (${\bar{\mathrm{AT}}}^{j})$, standard deviation (sd^*j*^), and standard error $(se_{\bar{j}}).$

		1S‐EP			2S‐EP			TM		
	*n*	${\bar{\mathrm{AT}}}^{\mathrm{EP}1}$	sd^EP1^	$se_{\bar{\mathrm{EP}1}}$	${\bar{\mathrm{AT}}}^{\mathrm{EP}2}$	sd^EP2^	$se_{\bar{\mathrm{EP}2}}$	${\bar{\mathrm{AT}}}^{\mathrm{TM}}$	sd^TM^	$se_{\bar{\mathrm{TM}}}$
PVEP	10	102	22.66	7.17	81.8	13.22	4.18	153.3	46.2	14.61
SEP	10	235.2	57.17	18.08	233.2	33.06	10.46	296.4	56.43	17.84
IONM	10	240	26.49	8.38	235	39.04	12.35	305.8	45.3	14.32

Abbreviations: IONM, intraoperative neurophysiological monitoring; PVEP, pattern visual evoked potential; SEP, somatosensory evoked potentials.

A time reduction of over 20% (with respect to TM) was achieved for SEP and IONM when the EPlacement devices were used. Note that this time reduction would be greater if the TM system had used all the steps required by the 10/20 system rather than the approximate method (ACP). For PVEP, a time reduction of over 30% was achieved with the new devices. In other words, when EPlacement was used, a time reduction of almost 1 min with respect to the traditional method was achieved for each test.

**FIGURE 5:**
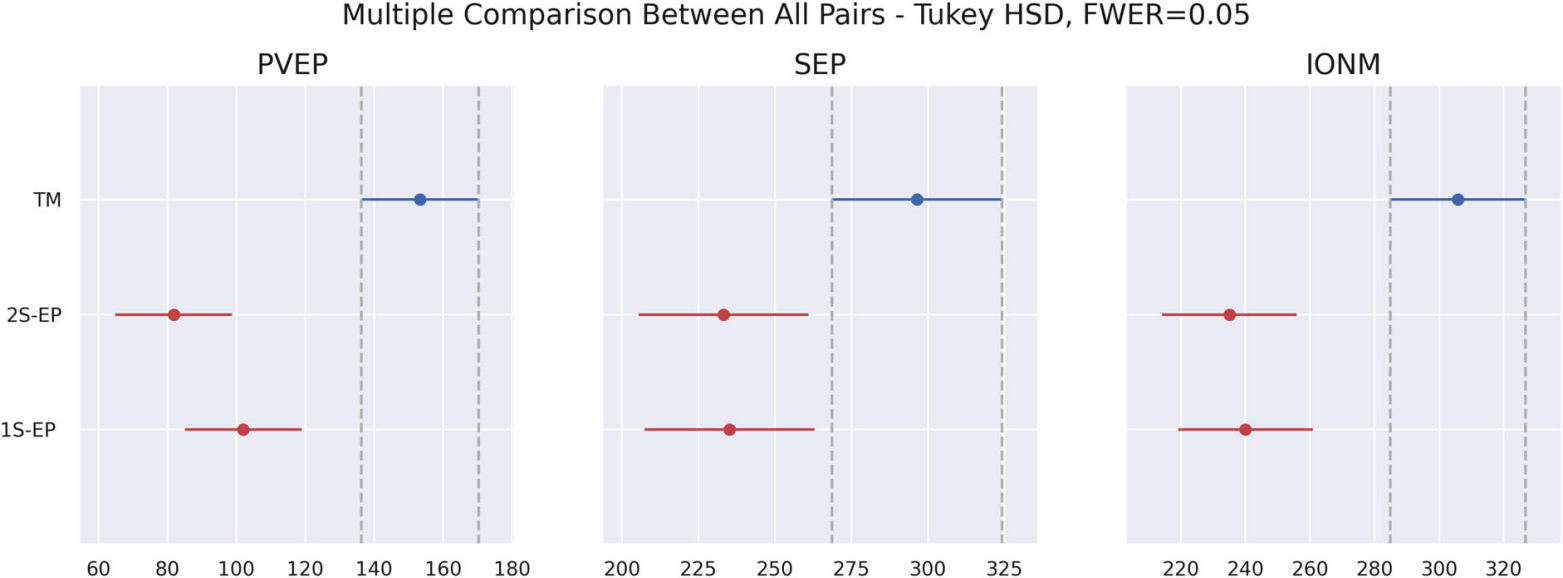
Tukey HSD test of times taken for the tools (tape measure [TM], 1S‐EP, and 2S‐EP) and neurophysiological tests (pattern visual evoked potential [PVEP], somatosensory evoked potentials [SEP], and intraoperative neurophysiological monitoring [IONM]).

To evaluate the significance of these time differences, Figure 5 shows the multiple comparison between all pairs (Tukey HSD test).

For the three tests analyzed (SEP, PVEP, and IONM), we can see that while no statistical differences appear between 1S‐EP and 2S‐EP, the mean time required for TM is always significantly greater.

### 3.3 Clinicians’ opinions

**FIGURE 6:**
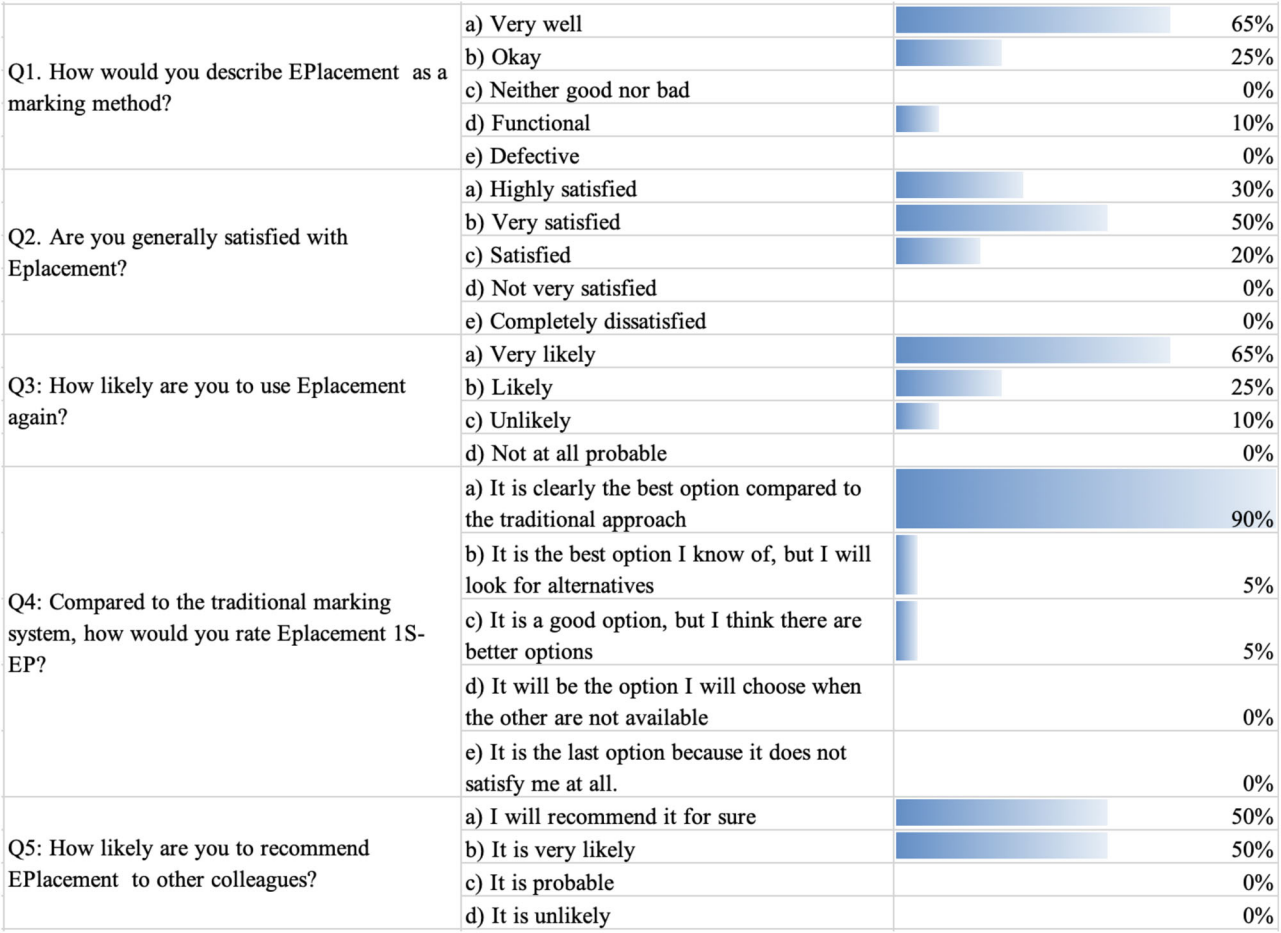
Results of the survey on EPlacement, completed by clinicians after marking the tests (pattern visual evoked potential [PVEP], somatosensory evoked potentials [SEP], intraoperative neurophysiological monitoring [IONM], and SEP) on volunteers.

**FIGURE 7:**
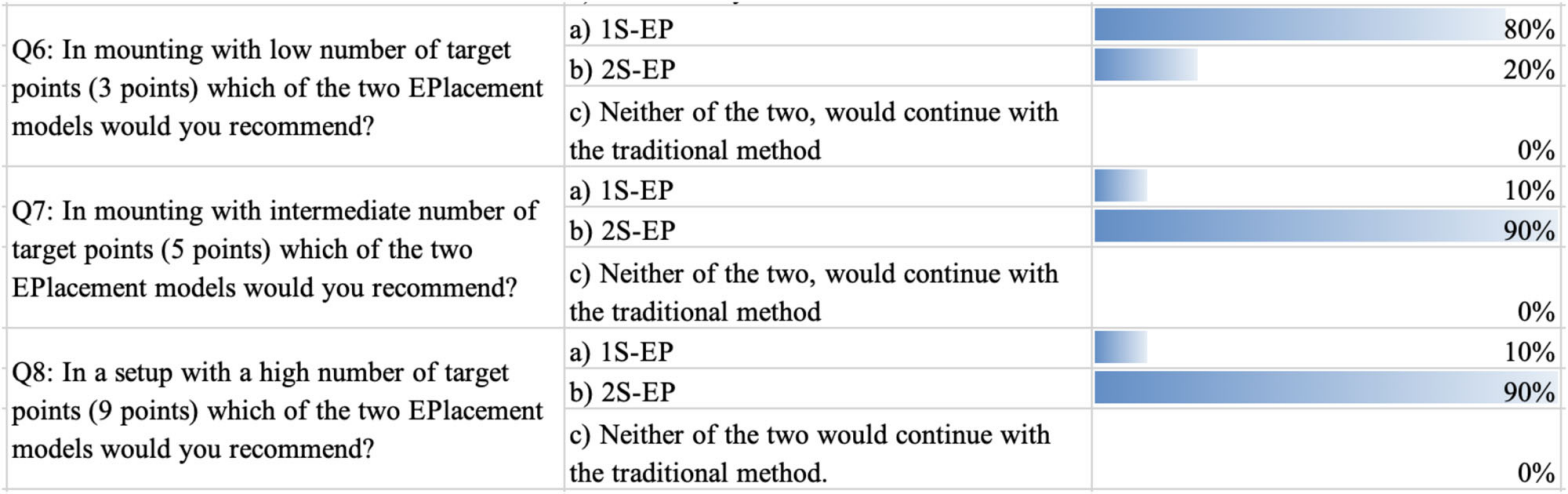
Results of the survey on marking with different numbers of electrodes, completed by clinicians after marking the tests (pattern visual evoked potential [PVEP], somatosensory evoked potentials [SEP], intraoperative neurophysiological monitoring [IONM], and SEP) on volunteers.

The survey shows that the clinicians were satisfied with the new method provided by the EPlacement devices. The survey was divided into two sections. In the first section, the clinicians expressed their opinions on the EPlacement device (Figure 6) and in the second section they expressed their opinions on the markings with different numbers of electrodes (Figure 7).

We found that 80% of clinicians reported that they were very satisfied or highly satisfied with 1S‐EP and 2S‐EP. As many as 90% of them also reported that if the device were purchased, they would be likely to use it and that EPlacement is clearly better than the TM method (90%). They also reported that they are very likely to recommend EPlacement to their colleagues.

The clinicians also reported that if the number of electrodes is less than five, and especially if all electrodes are in the same plane, they prefer version 1S‐EP. However, if the number of electrodes is five or more and the electrodes are in different planes, they prefer version 2S‐EP.

## 4 DISCUSSION

With regard to marking accuracy, we have clearly shown that errors made with the TM are much higher than those made with EPlacement and that errors made with both versions of EPlacement are similar. The errors made with EPlacement are similar to those reported by Xiao et al. (2017), who obtained an average error of 2.93 mm for the positions found in the NITT planes when using a navigational method. The above authors also reported an error of 6.41 mm for the manual measurement, which is close to the average error of 5 mm obtained in our study.

The results of our study are also similar to those reported in studies that used techniques such as MRI‐based electrode detection and obtained mean error values of 3.5 mm (Koessler et al., 2009). Dalal et al. (2014) found that the mean deviation of FlyTri electrode positions (3D MRI reconstructions using the 3D sensor) recorded with respect to the fringe projection was 1.5 mm, with a range between 0.5 and 2.9 mm (Dalal et al., 2014). Finally, in terms of positional errors, it has been shown that the mean error across all positions was 5.6 mm for the mri2mesh head models and 4.9 mm for the headreco head models (Guilherme et al., 2019), which indicates good accuracy. Our results with manual methods are therefore aligned with those of previous studies, and our results with EPlacement are similar to those of navigational methods.

These results illustrate the potential of this new device, since errors close to those of navigational methods have been obtained using a tool that is affordable for the vast majority of hospitals rather than with expensive, complex, and time‐consuming navigation systems.

With regard to the time spent performing the tests, no significant time differences were detected between EPlacement versions 1S‐EP and 2S‐EP. TM measurement, on the other hand, is much more time‐consuming because the 10/20 reference points must be measured in a sequence governed by the rules of the 10/20 system. These results are also aligned with those of the study by Xiao et al. (2017) from which TM times can be calculated as 3′ 30″ (three electrodes), 5′ 05″ (five electrodes), and 6′ 57″ (nine electrodes), and navigation system times can be calculated as 2′ 54″ (three electrodes), 3′ 49″ (five electrodes), and 4′ 40″ (nine electrodes). The times measured in the present study, therefore, verify the similarity of manual methods and confirm that EPlacement times are similar to those of navigation methods.

EPlacement enables clinicians to avoid taking measurements, making calculations, and memorizing the 10/20 rules, thus reducing their burden and lowering the possibility of error.

The difference between the two versions of EPlacement was small because the analyses were conducted on a mannequin. However, on the head of a patient with hair, version 2S‐EP would be faster since, unlike version 1S‐EP, it does not require the clinician to look for prior markings on their hair (which are difficult to identify).

Our survey results show that EPlacement makes marking easier for health care professionals. This is thanks to the step‐by‐step guidance provided with the device, which enables unskilled staff to perform the complex task of marking.

One limitation of the study is the use of mannequins instead of real human heads. Conducting the entire study on real human heads would have introduced confounding factors related to the accuracy of the new positioning device due to variations in individual head morphology, including skull shape and cranial landmark differences. However, the use of mannequins allowed for control over these factors, enabling a focused evaluation of the device's functionality and performance in a controlled setting. Although the part of the study with real human heads did not include quantitative measurements, the collection of subjective feedback from clinicians provided valuable insights into the device's usability in real‐life scenarios. Recognizing the subjective nature of the data collected, future research could address these limitations by incorporating a more quantitative analysis across diverse clinical cases.

## 5 CONCLUSIONS

Our results show that this new method based on the EPlacement device represents an improvement on conventional TM marking and may be considered within the group of advanced methods such as navigation systems since it leads to improvements of 34% (1.7 mm) for electrode positions in the Ns‐In and LPA–RPA lines (NITT) and 77% (12.5 mm) for electrode positions using the approximate method (ACP). This significant result highlights the current deficiency of marking at positions that are not on the Ns‐In or LPA–RPA lines. As well as improving accuracy, the EPlacement devices reduce the time spent per patient by an average of 1 min per test. Finally, in the survey the clinicians clearly showed that they are willing to use the new device in routine clinical practice.

### PEER REVIEW

The peer review history for this article is available at https://publons.com/publon/10.1002/brb3.3187.

## Data Availability

The data that support the findings of this study are available from the corresponding author, [A. Fabregat‐Sanjuan], upon reasonable request.
